# Mycotic aneurysm of the posterior tibial artery – a rare complication of bacterial endocarditis: a case report

**DOI:** 10.1186/1752-1947-2-341

**Published:** 2008-11-06

**Authors:** S Patel, N D'Souza, SV Gurjar, JC Hewes, W Edrees

**Affiliations:** 1 Department of Surgery, Medway Maritime Hospital, Gillingham, Kent, ME7 5NY, UK

## Abstract

**Introduction:**

Distal arterial embolisation and subsequent aneurysm formation are rare occurrences and most are secondary to trauma. We have found no case reports that describe posterior tibial aneurysm formation secondary to bacterial endocarditis.

**Case presentation:**

We report the case of a 47-year-old Caucasian man who, 2 years after an episode of subacute bacterial endocarditis, presented with signs and symptoms consistent with posterior tibial aneurysm formation.

**Conclusion:**

Posterior tibial aneurysm formation is a rare occurrence, most commonly occurring after trauma and, although other causes have been described, to our knowledge, endocarditis has not been implicated before, and as such should therefore be borne in mind when dealing with cases where no obvious aetiology is evident.

## Introduction

An aneurysm is a localised permanent dilatation of an artery greater than 50% of its expected normal diameter [1] – its formation can be attributed to various causes. Posterior tibial artery aneurysms are most often the result of traumatic injury to the artery; atraumatic aneurysms are rare and are usually thought to be of degenerative or unknown aetiology. This article describes a patient who developed a posterior tibial artery aneurysm secondary to bacterial endocarditis and its embolic sequelae.

## Case presentation

**Figure 1:**
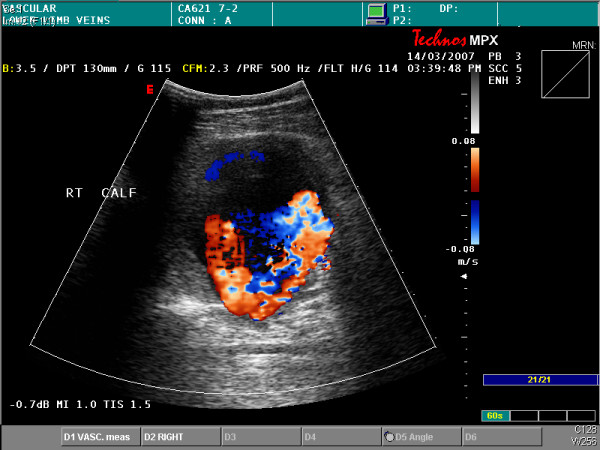
Colour duplex sonography showing a large aneurysm in the posterior tibial artery.

**Figure 2:**
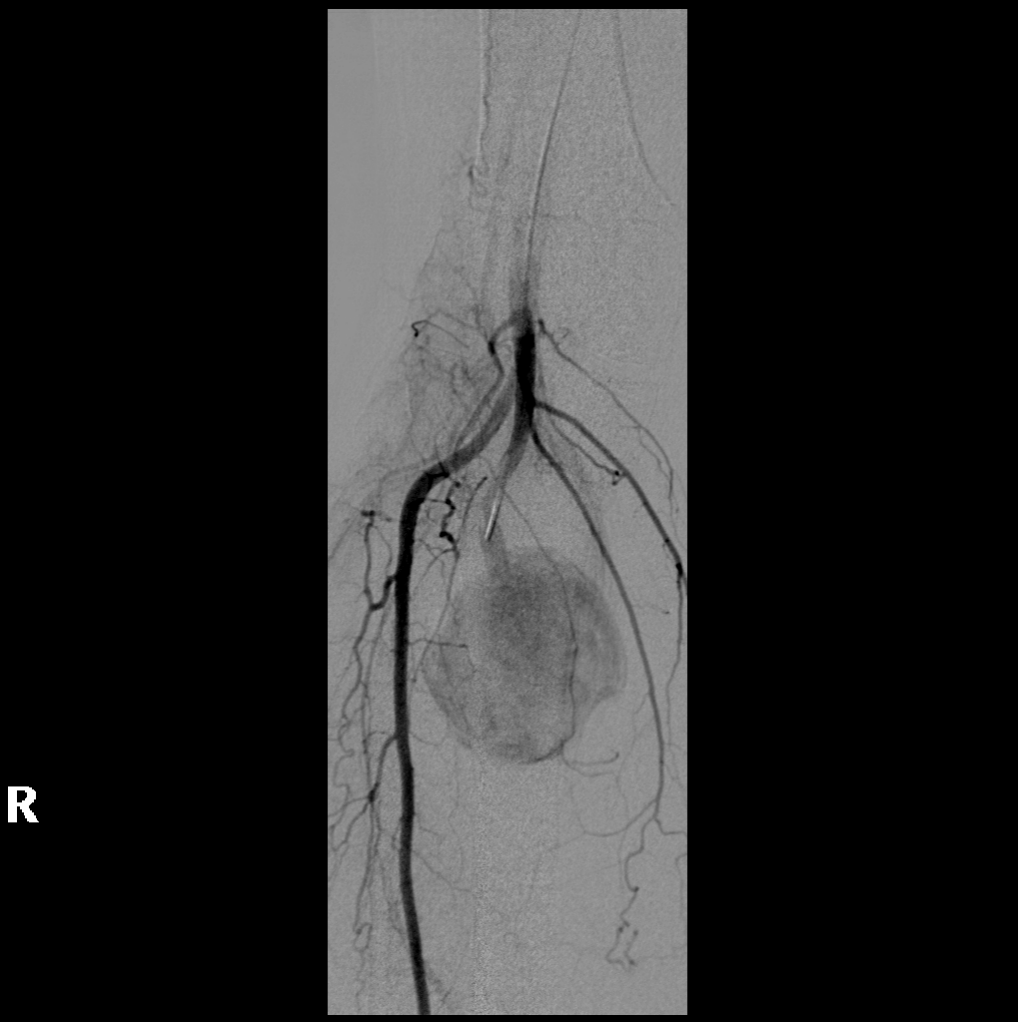
**Lower limb angiogram showing the presence of a large aneurysm in the posterior tibial artery with normal surrounding leg arteries and branches.** No angiographical evidence of atherosclerotic disease.

**Figure 3:**
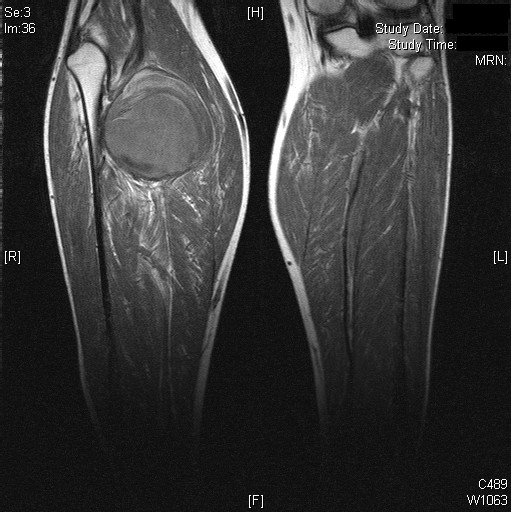
Bilateral magnetic resonance angiograms delineating the anatomy of the aneurysm and some surrounding tissue structures.

A 47-year-old Caucasian man presented with a 1-year history of worsening right calf swelling, claudicant pain and foot numbness. Examination revealed an 8 cm aneurysmal swelling in the lower popliteal fossa: a posterior tibial artery aneurysm was confirmed on ultrasonography, angiography and magnetic resonance imagery (Figures 1, 2, 3). The popliteal, anterior tibial and peroneal arteries were normal. There was no evidence of distal embolic pathology. The patient gave no relevant history of atherosclerosis, inflammatory arteritis or traumatic injury. He had however been treated 2 years before this presentation for subacute bacterial endocarditis following dental extractions. There had been confirmed radiographic evidence of mycotic embolisation to both kidneys and spleen; mitral valve vegetations were demonstrated on echocardiography. The patient was treated conservatively with an appropriate antibiotic regime, and his symptoms and elevated inflammatory indices eventually resolved.

**Figure 4:**
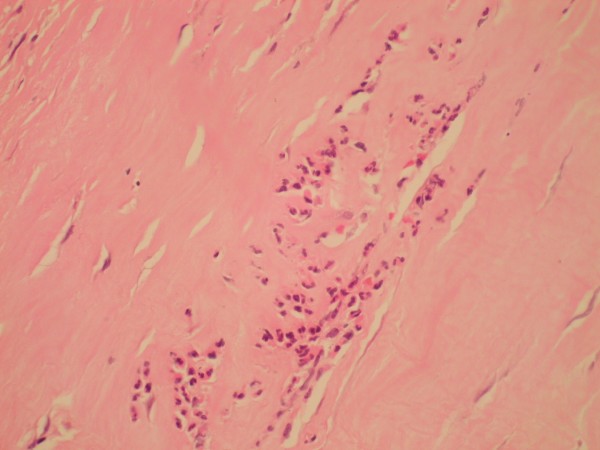
Aneurysm wall showing fibrosis, loss of elastic tissue and a neutrophil polymorph infiltrate.

The patient underwent elective surgical repair of the posterior tibial artery aneurysm with a reversed short saphenous vein jump graft. Postoperatively, he made an uneventful recovery. Histology showed pieces of aneurysm wall with attached skeletal muscle and thrombus. The aneurysm wall was fibrotic, with destruction of elastic tissue and an infiltrate of neutrophil polymorphs, consistent with an infective process (Figure 4).

The posterior tibial artery aneurysm was considered to be mycotic and related to the previous history of endocarditis.

## Discussion

Atraumatic aneurysms of the posterior tibial artery are rare with only 11 isolated case reports in the literature. The majority of these were described as either idiopathic or degenerative in origin.

Osler first described mycotic aneurysm formation in 1885. It is recognised to be the result of an infected embolus (usually vegetative) lodging within an artery leading to an exudative mesarteritis, and subsequent partial digestion of elements of the arterial wall. The eventual result is focal mural necrosis and subsequent aneurysm formation [2].

Embolism may occur in 22% to 50% of patients with endocarditis, usually resulting in arterial occlusion rather than aneurysm formation; emboli are most likely to lodge at arterial branch points [3]. Mycotic aneurysms occur most frequently in the intracranial arteries (65%), followed by visceral arteries and vessels of the upper and lower limbs. The rate of embolism falls after the first 3 weeks of antimicrobial therapy, although it can still occur after therapy is completed [4]. Valvular surgery has a role in the prevention of embolism with the greatest benefit being seen in the early stages of the disease. Patients with large vegetations, recurrent embolism, antibiotic-resistant organisms, and prosthetic valves are at particularly high risk of embolic phenomena [4].

Management of mycotic aneurysm depends on its size and location although surgery is the mainstay of treatment. The artery may be embolised angiographically or ligated surgically if sufficient distal blood flow can be maintained [5]. Percutaneous occlusion with thrombin has also been described [6].

Bypass procedures are complicated by the inherent presence of a septic focus and/or perivascular inflammation: prosthetic grafts should therefore be avoided. Autologous vein graft can be used to bypass an excised aneurysm although infection related graft failure remains a significant complication. Extra-anatomical bypass through uninfected tissue planes may avoid this. The patient may have to remain on long-term antibiotics.

## Conclusion

There was no obvious precipitating cause for the aneurysm identified in this patient. He did not suffer from atherosclerotic disease and had no vasculitic disorders or trauma to the region. We suggest that this unusual aneurysm originated from an embolic vegetation that had settled on the vessel wall and caused erosion and subsequent mural weakness over the 2 years before presentation. In the absence of this patient's previous history of endocarditis and embolic phenomena, it would probably have been thought to be of an idiopathic aetiology. In patients with seemingly idiopathic aneurysms, investigation for possible sources of vegetative emboli should therefore be undertaken.

## Consent

Written informed consent was obtained from the patient for publication of this case report and any accompanying images. A copy of the written consent is available for review by the Editor-in-Chief of this journal.

## Competing interests

The authors declare that they have no competing interests.

## Authors' contributions

SP drafted and edited the manuscript. ND'S, SG and JH helped draft the manuscript. WE performed the surgery. All authors read and approved the final manuscript.
